# Potential relevance of pre-operative quality of life questionnaires to identify candidates for surgical treatment of genital prolapse: a pilot study

**DOI:** 10.1186/1471-2490-12-9

**Published:** 2012-03-27

**Authors:** Christian Chauvin, Elisabeth Chéreau, Marcos Ballester, Emile Daraï

**Affiliations:** 1 Service de Gynécologie-Obstétrique, Hôpital Tenon, Assistance Publique des Hôpitaux de Paris, Université Pierre et Marie Curie Paris 6, Paris, France

**Keywords:** Genital prolapse, Quality of life questionnaire, Surgery

## Abstract

**Background:**

To evaluate prolapse-related symptoms, quality of life and sexuality of patients with validated questionnaires before and after surgery for genital prolapse and assess relevance of such an evaluation to select women for surgery.

**Methods:**

From November 2009 to April 2010, 16 patients operated on for genital prolapse of grade greater than or equal to 2 (POP-Q classification) were evaluated prospectively by three questionnaires of quality of life Pelvic Floor Distress Inventory (PFDI-20), Pelvic Floor Impact Questionnaire (PFIQ-7) and Pelvic Organ Prolaps/Urinary Incontinence Sexual Questionnaire (PISQ-12). Data were collected the day before surgery and 6 weeks postoperatively.

**Results:**

Eleven patients had laparoscopic surgery and five vaginal surgery. There was a significant decrease in pelvic heaviness, vaginal discomfort and urinary symptoms after surgery. The score of symptoms of prolapse, the PFDI-20 score was 98.5 preoperatively and 31.8 postoperatively (p < 0.0001). The score for quality of life, the PFIQ-7 score was 54.5 preoperatively and 7.4 postoperatively (p = 0.001). The score of sexuality, the PISQ-12 score was 35.3 preoperatively and 37.5 postoperatively (p = 0.1). Two of the 3 patients with a PFIQ 7 under or equal to 20 were not improved while all the women with a preoperative PFIQ-7 over 20 were improved after surgery.

**Conclusions:**

This study suggests that surgery improves quality of life of patients with genital prolapse. Quality of life questionnaires could help select good candidates for surgery. Further studies are required to determine threshold to standardize indications of surgery.

## Background

Genital prolapse, also known as pelvic organ prolapse (POP), is a major public health issue in western populations where as many as 38% to 76% of women consulting for routine gynaecological care suffer from the condition [1]. The lifetime risk of these women undergoing genital prolapse surgery is estimated at 11.8% [2]. Patients referred for surgery frequently complain about bulging and associated urinary, bowel or sexual symptoms, which are responsible for a significant decrease in health-related quality of life [3-8].

The decision criteria for surgery are mainly based on anatomical abnormalities [3,5]. However, there is no objective argument to correlate anatomical abnormalities with the impact on quality of life of patients with genital prolapse. In other disorders, it has been shown that the evaluation of symptoms or discomfort by analogue scales did not correlate with changes in quality of life after surgery [9]. Several quality of life questionnaires have been validated to evaluate changes after surgery for genital prolapse, but none of them have been used to clarify indications for surgery [10-17]. Hence, it would be useful to have predictors of changes in quality of life after treatment of genital prolapse to identify good candidates for this functional surgery.

The objectives of this pilot study were to evaluate the impact of surgery on quality of life in women with genital prolapse using validated quality of life questionnaires and to assess whether these questionnaires could be useful tools to select patients for surgery.

## Methods

We conducted a pilot study from November 2009 to April 2010 on prospective data of patients undergoing genital prolapse repair. Women either underwent laparoscopic sacrocolpopexy (LSC) or surgery by vaginal route using synthetic or biological mesh. The latter approach was recommended in patients with co-morbidities contraindicating the laparoscopic approach such as severe respiratory failure, morbid obesity or prior multiple surgery by laparotomy. Among the 16 patients included in this pilot study, 11 underwent an LSC and five a vaginal route. The decision to include sling in the procedure was done according to clinical and urodynamic stress incontinence.

Preoperative and follow-up pelvic examinations to evaluate genital prolapse stage used the International Continence Society pelvic organ prolapse quantification system (POPQ) [18]. The maximal extent of prolapse was clinically measured during a Valsalva maneuver or coughing and was confirmed by the patient as being the most severe protrusion. Anatomical recurrence was defined POPQ stage ≥ II (≥ -1 cm). All patients gave written informed consent to participate in this pilot study. Ethical Committee approved this pilot study.

### Surgical techniques

For LSC, lightweight macroporous polypropylene mesh (Parietex^® ^Covidien, USA) was used. All patients were operated on under general anaesthesia. After CO2 intra-peritoneal insufflation at 12 mmHg with a Veress needle, a 12 mm trocar was inserted at the umbilicus for the scope, two 5 mm trocars at the right and left iliac fossae, and a suprapubic incision made for a 15 mm trocar. The first step of the procedure consisted of a subtotal hysterectomy with bilateral oophorectomy for postmenopausal patients if the uterus was still in place. Vesicovaginal cleavage was extended to the lower third of the vagina. The uterus and adnexes were extracted using an electric morcellator. The second step of the procedure began by continuing the rectovaginal dissection to the lower third of the posterior vaginal wall and then extending it laterally to visualize the levator ani muscle fascia. Next, the peritoneum facing the sacral promontory was opened to visualize the anterior vertebral ligament. The peritoneal opening was extended downwards so as to join the rectovaginal dissection. The posterior mesh was secured to the levator ani muscle fascia using nonabsorbable sutures or staples. The anterior mesh was then secured to the anterior vaginal wall using three nonabsorbable sutures. Finally, the anterior and posterior meshes were secured to the anterior vertebral ligament at the sacral promontory using a nonabsorbable suture or staples before closing the peritoneum with absorbable sutures. After exsufflation, the skin incisions were closed with absorbable sutures as well. A Foley catheter was left in place for 24 hours.

For the vaginal route a mixed mesh composed of polypropylene and porcine dermis was used (Avaulta^® ^implant from Baxter, USA). Surgery was performed with the patient in the dorsal lithotomy position under general or spinal anaesthesia. Genital prolapse repair was preceded by vaginal hysterectomy. If the latter had already been performed, an incision was made at the vaginal apex. The vaginal mucosa was separated from the bladder by dissection. After incising the perineal skin facing the obturator membrane, the endopelvic fascia was perforated with Avaulta kit from the skin to the incised vaginal wall according to laboratory recommendations [19]. The same procedure was performed on the contralateral side. Then approximation of the mesh under the urethra and bladder was performed. Finally, the colpotomy and skin incisions were closed with absorbable sutures. A Foley catheter was left in place for 24 hours.

Follow-up pelvic examination was carried out 4 to 6 weeks after surgery, then once every six months up to date. Patients were asked to answer validated quality of life questionnaires at the preoperative visit and then at each follow-up visit or through telephone interviews by investigators who were blinded to the type of surgery. The short version of the Pelvic Floor Distress Inventory (PFDI-20), the Pelvic Floor Impact Questionnaire (PFIQ-7) and Pelvic Organ Prolaps/Urinary Incontinence Sexual Questionnaire (PISQ-12) were used [13-15].

The PFDI-20 assesses the presence and amount of distress caused by 20 symptoms related to pelvic floor disorders. It is composed of three sub-questionnaires; the Pelvic Organ Prolapse Distress Inventory (POPDI-6) which includes 6 items, the Colon Rectal Anal Distress Inventory (CRADI-8) which includes 8 items and the Urinary Distress Inventory (UDI-6) which includes 6 items. Patients were asked if they experienced each symptom, and if so, how much the symptom bothered them on a scale of 1 (not at all) to 4 (severe). Scores for each sub-questionnaire range from 0 to 100 with higher scores indicating greater symptom distress.

The PFIQ-7 assesses the impact of symptoms on activities of daily living. It is composed of three sub-questionnaires consisting of 7 items each; the Pelvic Organ Prolapse Impact Questionnaire (POPIQ-7), the Colon Rectal Anal Impact Questionnaire (CRAIQ-7) and the Urinary Impact Questionnaire (UIQ-7). Scores for each sub-questionnaire range from 0 to 100 with higher scores indicating greater symptom distress.

The PISQ-12 assesses the impact of symptoms on sexual satisfaction and includes 12 items scored from 0 to 4. Total scores range from 0 to 48 with higher scores indicating greater sexual satisfaction.

### Statistical analysis

Statistical analyses were carried out using the R 2.11^® ^software. Qualitative variables were compared using the Fisher's exact test or Chi2 test, and quantitative variables by the Wilcoxon rank-sum test. A p value of 0.05 denoted a significant difference.

## Results

### Epidemiological and surgical characteristics of the patients

The mean age of the patients was 63 years (range: 42-88) and the mean body mass index (BMI) was 25.5 kg/m2 (range: 17.9-32.7). The mean parity was 3 (range: 2-6). A history of surgery for urinary incontinence was noted in one patient and a previous operation for genital prolapse in another. Eleven of the 16 patients were menopausal including one patient on hormone replacement therapy.

Twelve of the 16 patients underwent a hysterectomy including seven with bilateral salpingo-oophorectomy. Among the four remaining patients, three had conservative surgery and one had underwent previously hysterectomy for fibroma. Three patients had stress urinary incontinence treated by a suburethral sling procedure via the transobturator route. One of the five patients undergoing vaginal route underwent a bilateral sacrospinous fixation. None of the patients had a posterior colpoperineorrhaphy.

The mean drop in hemoglobin was 1.5 g/dl (range: 0.1-3.5). No patient required a blood transfusion. No bowel, bladder or ureteral injury was observed. The mean hospital stay was 2.3 days (range: 2-5).

### Changes in symptoms and anatomical results after surgical treatment for genital prolapse

A significant decrease in pelvic pain was observed (13 patients preoperatively vs one postoperatively) (p < 0.01) and in vaginal discomfort (15 patients preoperatively vs none postoperatively) (p < 0.01). No patient reported *de novo *pelvic pain or vaginal discomfort. No difference in urinary dysfunction was observed: two patients experienced pollakiuria preoperatively vs none postoperatively; four patients experienced nocturia preoperatively vs none postoperatively; and three patients experienced urgency preoperatively vs two postoperatively. No patients reported *de novo *urinary symptoms, anorectal symptoms or pre-or postoperative constipation. One patient reported pain on defecation preoperatively as opposed to none postoperatively.

At the first postoperative visit, 15 patients had optimal anatomic results and one operated on via the vaginal route had persistent asymptomatic posterior prolapse (-2 cm).

The median follow-up was 16 months (range: 14-19). No recurrence of genital prolapse and no mesh infection or rejection was observed during follow-up whatever the surgical route.

### Changes in quality of life

Changes in quality of life assessed by PFDI-20, PFIQ-7 and PISQ-20 questionnaires Pelvic Floor Distress Inventory (PFDI-20), Pelvic Floor Impact Questionnaire (PFIQ-7) and Pelvic Organ Prolaps/Urinary Incontinence Sexual Questionnaire (PISQ-12) are summarized in Table 1.

**Table 1:** Quality of life evaluated by the PFDI-20, PFIQ-7 and PISQ-12 questionnaires before and after surgery for genital prolapsed

	Preoperative values Median (range)	Postoperative values Median (range)	P value
**PFDI-20**	98.5 (33-235)	31.8 (0-103)	**< 0.0001**

POPDI-6	46.1 (8-100)	10.1 (0-33)	**0.0004**

CRADI-8	23.5 (0-79)	12.5 (0-53)	**0.01**

UDI-6	30.4 (0-91)	9.1 (0-33)	**0.001**

**PFIQ-7**	54.5 (0-162)	7.4 (0-43)	**0.001**

UIQ-7	25.3 (0-76)	3 (0-19)	**0.009**

CRAIQ-7	11.6 (0-52)	3.6 (0-43)	**0.03**

POPIQ-7	17.6 (0-52)	0.9 (0-14)	**0.003**

**PISQ-12**	35.3 (27-41)	37.5 (30-46)	0.1

The PFDI-20 questionnaire revealed a decrease in symptoms related to prolapse (p < 0.0001). This decrease was significant for the three sub-questionnaires, the POPDI-6 (Pelvic Organ Prolapse Distress Inventory) (p < 0.0004), the CRADI-8 (Colon Rectal Anal Distress Inventory) (p = 0.01) and UDI-6 (Urinary Distress Inventory) (p = 0.001). Analysis of the PFDI-20 scores per patient showed that all the 16 patients were improved by surgery.

An overall significant improvement in quality of life was shown by the PFIQ-7 scores (p = 0.001): the POPIQ-7 (Pelvic Organ Prolapse Impact Questionnaire) (p = 0.009), the CRAIQ-7 (Colon Rectal Anal Impact Questionnaire) (p = 0.03) and the UIQ-7 (Urinary Impact Questionnaire) (P = 0.003). Analysis of the PFIQ-7 scores per patient showed that the 13 patients with a preoperative PFIQ-7 above 20 were improved by surgery while two of the three patients with a preoperative PFIQ-7 under or equal to 20 reported no improvement after surgery. These patients were elderly patients with mean age of 78 years with predominant anterior prolapse and two of them were operated on via the vaginal route.

The PISQ-12 scores did not show improvement in sexual quality of life after surgery (p = 0.1).

## Discussion

The present pilot study demonstrates that quality of life of patients with advanced stage of genital prolapse was improved by surgery and indicates that the PFIQ-7 is the most appropriate questionnaire to identify patients that could benefit from surgery.

The aim of genital prolapse surgery is not only anatomical correction but also to improve functional symptoms and quality of life. A recent Cochrane review of the surgical management of genital prolapse noted that the impact of surgery on associated pelvic floor symptoms and quality of life were poorly reported explaining why it is difficult to identify good candidates for this functional surgery [20]. After failure of perineal rehabilitation and the use of pessaries, current recommendations suggest that surgery should be an option for women with advanced stages of genital prolapse. But the use of exclusive anatomical criteria seems questionable as no strict relation exists between anatomical correction and improvement in symptoms and quality of life [21,22]. Visual analogue scales assessing the intensity of symptoms associated with genital prolapse could be used but to date there are no guidelines as to the threshold at which surgery is recommended. Moreover, particularly for patients with endometriosis, a low correlation between evaluation of symptoms by visual analogue scale and changes in quality of life after surgery has been shown [23]. On the other hand, the usefulness of quality of life questionnaires for selecting patients who could derive benefit from surgery has already been demonstrated [23]. Recently, several reports have demonstrated the very good psychometric properties of quality of life questionnaires to evaluate the impact of genital prolapse with a Cronbach's alpha greater than 0.70 for all items, confirming that questionnaires are able to detect changes in quality-of-life after treatment [24,25]. However, to our knowledge, no study has attempted to assess the contribution of quality of life questionnaires specific to genital prolapse to identify good candidates for surgery.

Analysis of the pre- and postoperative values of the PFDI-20 showed that all 16 patients of this pilot study experienced an improvement in quality of life. These results are consistent with those of previous reports showing the positive impact of genital prolapse surgery [26]. In a review of the literature on laparoscopic treatment of genital prolapse, Ganatra et al. reported a patient satisfaction of 94.4% with a median follow-up of 24.6 months [26]. In addition, our data are consistent with those of retrospective studies demonstrating a significant improvement in quality of life after surgical cure of genital prolapse by both vaginal route and laparoscopy [22,26-31]. Moreover, unlike retrospective series, our study shows an improvement in anorectal symptoms assessed by the sub-questionnaire CRADI-8 [32]. Similar results were noted using the PFIQ-7 questionnaire. In this pilot study, even if all the patients had advanced genital prolapse stages, a wide spectrum of quality of life questionnaire scores was observed. Scores from the PFDI-20 questionnaire ranged from 33 to 235 (on a scale of 0 to 300). Similarly, the preoperative PFIQ-7 questionnaire scores ranged from 0 to 162. It is important to note that some patients did not report impairment in quality of life related to genital prolapse. The apparent low impact of genital prolapse on sexual quality of life observed in this study suggests that the PISQ-12 questionnaire is not a useful tool to select candidates for genital prolapse surgery. This is in agreement with previous studies showing no improvement or deterioration in the sexual quality of life after genital prolapse cure [21,22]. Moreover, the PISQ-12 questionnaire can only be used for sexually active patients.

However, even if postoperative improvement in quality of life has been demonstrated in series of patients, it remains difficult to predict impact on an individual basis. To date, no nomogram or recursive partitioning model to select patients for surgery has been developed. Our results are too preliminary to determine which questionnaire could be used to construct a nomogram predicting good outcome after surgery. However, the PFDI-20 questionnaire has the advantage of giving a wide distribution of preoperative values but with a ratio of pre-over postoperative mean values of 3 (98.5 preoperatively vs 31.8 postoperatively). The PFIQ-7 questionnaire gives preoperative values that are relatively scattered but the pre-over postoperative mean value ratio was 7 (54.5 vs 7.4) suggesting a higher power of discrimination compared to the PFDI-20. Moreover, the PFIQ-7 can identify patients who are not likely to benefit from surgery. Indeed, two of the three patients with a preoperative PFIQ-7 less than or equal to 20 had no improvement in quality of life after surgery while the remaining 13 patients with a preoperative PFIQ-7 above 20 were improved. Our results are in accordance with those of Lawndy et al. [33] showing that even if no difference was observed in anatomical results, some patients reported the absence of symptoms improvement.

This pilot study therefore gives some important pointers to calculate sample size for a study to build a predictive model of quality of life improvement after genital prolapse cure. Taking into account that two of the 16 patients had no improvement using the PFIQ-7 questionnaire and that at least 50 patients with no improvement are required to built a predictive model, at least 400 patients would be required for such a study.

The limitations of this pilot study should be highlighted. First, the low number of patients may be a potential source of bias. Despite this disadvantage, our pilot study underlined the wide spectrum of preoperative values to quality of life questionnaires in patients with genital prolapse. Even if all our patients had advanced genital prolapse stage, they represented a heterogeneous population underlining that anatomical abnormalities associated with genital prolapse are insufficient to select patients for surgery. Second, two routes for genital prolapse cure were used. It is possible that the type of surgery could influence changes in quality of life. Finally, the short follow-up cannot exclude the risk of overestimating the benefit of surgery.

## Conclusions

Despite the limits of this pilot study, our data indicate that quality of life questionnaires could be a useful tool to select patients for genital prolapse surgery. Further large studies are obviously required taking into account quality of life not only of patients with advanced genital prolapse stages but also the route of surgical management, to determine a threshold to standardize indications for surgery.

## Competing interests

The authors declare that they have no competing interests.

## Authors' contributions

Protocol/project development: ED/EC, Data collection or management: CC/MB, Data analysis: EC/MB/ED, Manuscript writing/editing: CC/EC/ED. All authors read and approved the final manuscript.

## Pre-publication history

The pre-publication history for this paper can be accessed here:

http://www.biomedcentral.com/1471-2490/12/9/prepub
